# Supplementary material for: The Influence of the Grapevine Bacterial and Fungal Endophytes on Biomass Accumulation and Stilbene Production by the In Vitro Cultivated Cells of Vitis amurensis Rupr
Source: Plants (Basel). 2021 Jun 23;10(7):1276. doi: 10.3390/plants10071276 (PMC8309151; doi:10.3390/plants10071276)
Supplement: Supplementary file 1 [file plants-10-01276-s001.zip › plants-1239276-supplementary.pdf]

Supplementary Table S1.

Primers used for amplification of *Vitis amurensis* cDNAs in real-time PCRs

cDNA (GeneBank)	Primers, 5'-3'
Primers for real-time PCR, 5'-3'	
VaPAL1 (EU659859)	5'CAG GAC TTC ACC TCA ATG G–3'<br>5'GGG TTA TCA TTA ACG GAG TTG A–3'
VaPAL2 (EU659860)	5'AAG GAC TTC CCC TCA ATG G–3'<br>5'GGG TTA TCG TTC ACT GAG TTT A–3'
VaPAL3 (EU659861)	5'TCC GAA CAT CTC CCC AGT–3'<br>5'ATC AAG GGA TTG TCG TTT ACC–3'
VaPAL4 (GQ443744)	5'AGC AAG ATC GGT ATG CTC T–3'<br>5'TCT ATG GAC TTC GTT GAT GT–3'
VaPAL5 (GQ443745)	5'GTT GAT GAA GCC TAA ACA AG–3'<br>5'CCA TGC GAA TGA CCT CAA T–3'
VaSTS1 (EU659862)	5'CCA ACC AAA GTC CAA GAT CA<br>5'CCT TCT AAC CGA TGT TTC AAG A
VaSTS2 (EU659863)	5'CGG TGC GGA TTA CAA ACT C<br>5'CAC CCT TGA TGG TAC AAC AT
VaSTS3 (EU659864)	S1: GTC AGC CTA AAT CGA AGA TCA C<br>A1: TCT TCT AAC AGA TGG TTC GAG G
VaSTS4 (EU659865)	S1: ACA ACC TCT GGT GTA GAA AT<br>A1:CTG ACC GAT GTT TCA AGG C
VaSTS5 (EU659866)	S1: CCT CGA ACC ATC TGT TAG AAG A<br>A1: TCT CCG CAA GAT CCT TAG C
VaSTS6 (EU659867)	S1: CCT CAG GTG TAG AAA TGC C<br>A1: CAT CAC TCT TCT AAC AGA TGG
VaSTS7 (EU659868)	S1: GCA GCA CTG AAG GCA CTT AA<br>A1: ATT TCT ACA CCT GAC GTT GT
VaSTS8 (GQ443746)	S1: GCC TCG AAC CAT CTG TCA<br>A1: AAG ATC CTT AGC TGT TCG A
VaSTS9 (GU266256)	S1: GGC AGC CTA AGT CCA AGA TTA<br>A1: AGT CTG CAC CGG GCA TTT C
VaSTS10 (JQ780328)	S1: GCA GGA GCA CGG GTT CT<br>A1: AGT CCA AAG CAA TTT CAG AA
Va-actin1 (DQ517935)	S1: GTA TTG TGC TGG ATT CTG GTG AT<br>A1: AGC AAG GTC AAG ACG AAG GAT AG
VaGapdh (XM_002263109)	S1: CAC TGA AGA TGA TGT TGT TTC C<br>A1: GCT ATT CCA GCC TTG GCA T

Characteristics of bacteria and fungi based on *16S rRNA* gene (bacteria) or internal transcribed spacers *ITS1* (fungi) sequences used in experiments isolated from *Vitis amurensis* grape microbiome.

The resulting nucleotide sequences were collected using the Staden Package program. The percent identity of the collected nucleotide sequences is determined using a specialized program NCBI BLAST (http://blast.ncbi.nlm.nih.gov; May 11, 2021), using the Nucleotide Blast (nucleotide - nucleotide BLAST) algorithm.

1. ***Agrobacterium* sp., MZ424738** (s1860-1,7,13,19), 1443 bp.
AGAGTTTGATCCTGGCTCAGAACGAACGCTGGCGGCAGGCTTAACACATGCAAGTCGAACGCATCGCAAGATGA
GTGGCAGACGGGTGAGTAACGCGTGGGAATCTACCCATCTCTGCGGAATAGCTCTGGGAAACTGGAATTAATACC
GCATACGCCCTACGGGGGAAAGATTTATCGGGGATGGATGAGCCCGCGTTGGATTAGCTAGTTGGTGGGGTAAA
GGCCTACCAAGGCGACGATCCATAGCTGGTCTGAGAGGATGATCAGCCACATTGGGACTGAGACACGGCCCAAA
CTCCTACGGGAGGCAGCAGTGGGGAATATTGGACAATGGGCGCAAGCCTGATCCAGCCATGCCGCGTGAGTGAT
GAAGGCCTTAGGGTTGTAAAGCTCTTTCACCGGTGAAGATAATGACGGTAACCGGAGAAGAAGCCCCGGCTAACT
TCGTGCCAGCAGCCGCGGTAATACGAAGGGGGCTAGCGTTGTTCGGAATTACTGGGCGTAAAGCGCACGTAGGC
GGATATTTAAGTCAGGGGTGAAATCCCGCAGCTCAACTGCGGAACTGCCTTTGATACTGGGTATCTTGAGTATGG
AAGAGGTAAGTGGAATTGCGAGTGTAGAGGTGAAATTCGTAGATATTCGCAGGAACACCAGTGGCGAAGGCGGC
TTACTGGTCCATTACTGACGCTGAGGTGCGAAAGCGTGGGGAGCAAACAGGATTAGATACCCTGGTAGTCCACGC
CGTAAACGATGAATGTTAGCCGTCGGGCAGTTGACTGTTCGGTGGCGCAGCTAACGCATTAAACATTCCGCCTGG
GGAGTACGGTCGCAAGATTAAAACTCAAAGGAATTGACGGGGGCCCGCACAAGCGGTGGAGCATGTGGTTTAAT
TCGAAGCAACGCGCAGAACCTTACCAGCTCTTGACATTCGGGGTTTGGGCAGTGGAGACATTGTCCTTCAGTTAG
GCTGGCCCCAGAACAGGTGCTGCATGGCTGTCGTCAGCTCGTGTCGTGAGATGTTGGGTTAAGTCCCGCAACGAG
CGCAACCCTCGCCCTTAGTTGCCAGCATTTGGTTGGGCACTCTAAGGGGACTGCCGGTGATAAGCCGAGAGGAAG
GTGGGGATGACGTCAAGTCCTCATGGCCCTTACGGGCTGGGCTACACACGTGCTACAATGGTGGTGACAGTGGG
CAGCGAGACAGCGATGTCGAGCTAATCTCCAAAAGCCATCTCAGTTCGGATTGCACTCTGCAACTCGAGTGCATGA
AGTTGGAATCGCTAGTAATCGCAGATCAGCATGCTGCGGTGAATACGTTCCCGGGCCTTGTACACACCGCCCGTCA
CACCATGGGAGTTGGTTTTACCCGAAGGCGCTGCGCTAACCGCAAGGGGGCAGGCGACCACGGTAGGGTCAGCG
ACTGGGGTGAAGTCGTAACAAGGTA

***Agrobacterium rubi*,** MN752429.1, 1443bp, Percent identity 99.17%
AGAGTTTGATCATGGCTCAGAACGAACGCTGGCGGCAGGCTTAACACATGCAAGTCGAACGCCCCGCAAGGGGA
GTGGCAGACGGGTGAGTAACGCGTGGGAATCTACCCAACCCTGCGGAATAGCTCTGGGAAACTGGAATTAATAC
CGCATACGCCCTACGGGGGAAAGATTTATCGGGGATGGATGAGCCCGCGTTGGATTAGCTAGTTGGTGGGGTAA
AGGCCTACCAAGGCGACGATCCATAGCTGGTCTGAGAGGATGATCAGCCACATTGGGACTGAGACACGGCCCAA
ACTCCTACGGGAGGCAGCAGTGGGGAATATTGGACAATGGGCGCAAGCCTGATCCAGCCATGCCGCGTGAGTGA
TGAAGGCCTTAGGGTTGTAAAGCTCTTTCACCGGTGAAGATAATGACGGTAACCGGAGAAGAAGCCCCGGCTAAC
TTCGTGCCAGCAGCCGCGGTAATACGAAGGGGGCTAGCGTTGTTCGGAATTACTGGGCGTAAAGCGCACGTAGG
CGGATATTTAAGTCAGGGGTGAAATCCCAGAGCTCAACTCTGGAACTGCCTTTGATACTGGGTATCTTGAGTATGG
AAGAGGTAAGTGGAATTGCGAGTGTAGAGGTGAAATTCGTAGATATTCGCAGGAACACCAGTGGCGAAGGCGGC
TTACTGGTCCATTACTGACGCTGAGGTGCGAAAGCGTGGGGAGCAAACAGGATTAGATACCCTGGTAGTCCACGC
CGTAAACGATGAATGTTAGCCGTCGGGCAGTTGACTGTTCGGTGGCGCAGCTAACGCATTAAACATTCCGCCTGG
GGAGTACGGTCGCAAGATTAAAACTCAAAGGAATTGACGGGGGCCCGCACAAGCGGTGGAGCATGTGGTTTAAT
TCGAAGCAACGCGCAGAACCTTACCAGCTCTTGACATTCGGGGTTTGGGCAGTGGAGACATTGTCCTTCAGTTAG
GCTGGCCCCAGAACAGGTGCTGCATGGCTGTCGTCAGCTCGTGTCGTGAGATGTTGGGTTAAGTCCCGCAACGAG
CGCAACCCTCGCCCTTAGTTGCCAGCATTTAGTTGGGCACTCTAAGGGGACTGCCGGTGATAAGCCGAGAGGAAG
GTGGGGATGACGTCAAGTCCTCATGGCCCTTACGGGCTGGGCTACACACGTGCTACAATGGTGGTGACAGTGGG
CAGCGAGACAGCGATGTCGAGCTAATCTCCAAAAGCCATCTCAGTTCGGATTGCACTCTGCAACTCGAGTGCATGA
AGTTGGAATCGCTAGTAATCGCAGATCAGCATGCTGCGGTGAATACGTTCCCGGGCCTTGTACACACCGCCCGTCA
CACCATGGGAGTTGGTTTTACCCGAAGGCGCTGCGCTAACCGCAAGGGGGCAGGCGACCACGGTAGGGTCAGCG
ACTGGGGTGAAGTCGTAACAAGGTA

2. ***Bacillus* sp., MZ424739** (s1860-2,8,14,20), 1489bp.

CATGGGCGGCGTGCTATACATGCAGTCGAGCGAATGGATTGAGAGCTTGCTCTCAAGAAGTTAGCGGCGGACGG
GTGAGTAACACGTGGGTAACCTGCCCATAAGACTGGGATAACTCCGGGAAACCGGGGCTAATACCGGATAATATT
TTGAACTGCATGGTTCGAAATTGAAAGGCGGCTTCGGCTGTCACTTATGGATGGACCCGCGTCGCATTAGCTAGTT
GGTGAGGTAACGGCTCACCAAGGCAACGATGCGTAGCCGACCTGAGAGGGTGATCGGCCACACTGGGACTGAGA
CACGGCCCAGACTCCTACGGGAGGCAGCAGTAGGGAATCTTCCGCAATGGACGAAAGTCTGACGGAGCAACGCC
GCGTGAGTGATGAAGGCTTTCGGGTCGTAAAACTCTGTTGTTAGGGAAGAACAAGTGCTAGTTGAATAAGCTGGC
ACCTTGACGGTACCTAACCAGAAAGCCACGGCTAACTACGTGCCAGCAGCCGCGGTAATACGTAGGTGGCAAGCG
TTATCCGGAATTATTGGGCGTAAAGCGCGCGCAGGTGGTTTCTTAAGTCTGATGTGAAAGCCCACGGCTCAACCG
TGGAGGGTCATTGGAAACTGGGAGACTTGAGTGCAGAAGAGGAAAGTGGAATTCCATGTGTAGCGGTGAAATGC
GTAGAGATATGGAGGAACACCAGTGGCGAAGGCGACTTTCTGGTCTGTAACTGACACTGAGGCGCGAAAGCGTG
GGGAGCAAACAGGATTAGATACCCTGGTAGTCCACGCCGTAAACGATGAGTGCTAAGTGTTAGAGGGTTTCCGCC
CTTTAGTGCTGAAGTTAACGCATTAAGCACTCCGCCTGGGGAGTACGGCCGCAAGGCTGAAACTCAAAGGAATTG
ACGGGGGCCCGCACAAGCGGTGGAGCATGTGGTTTAATTCGAAGCAACGCGAAGAACCTTACCAGGTCTTGACAT
CCTCTGAAAACCCTAGAGATAGGGCTTCTCCTTCGGGAGCAGAGTGACAGGTGGTGCATGGTTGTCGTCAGCTCG
TGTCGTGAGATGTTGGGTTAAGTCCCGCAACGAGCGCAACCCTTGATCTTAGTTGCCATCATTAAGTTGGGCACTC
TAAGGTGACTGCCGGTGACAAACCGGAGGAAGGTGGGGATGACGTCAAATCATCATGCCCCTTATGACCTGGGCT
ACACACGTGCTACAATGGACGGTACAAAGAGCTGCAAGACCGCGAGGTGGAGCTAATCTCATAAAACCGTTCTCA
GTTCGGATTGTAGGCTGCAACTCGCCTACATGAAGCTGGAATCGCTAGTAATCGCGGATCAGCATGCCGCGGTGA
ATACGTTCCCGGGCCTTGTACACACCGCCCGTCACACCACGAGAGTTTGTAACACCCGAAGTCGGTGGGGTAACCT
TTATGGAGCCAGCCGCCTAAGGTGGGACAGATGATTGGGGTGAAGTCGTAGCAAGTACCCTGAGGG

Bacillus thuringiensis, KU179338.1, 1489 bp., Percent identity 100%
CATGGGCGGCGTGCTATACATGCAGTCGAGCGAATGGATTGAGAGCTTGCTCTCAAGAAGTTAGCGGCGGACGG
GTGAGTAACACGTGGGTAACCTGCCCATAAGACTGGGATAACTCCGGGAAACCGGGGCTAATACCGGATAATATT
TTGAACTGCATGGTTCGAAATTGAAAGGCGGCTTCGGCTGTCACTTATGGATGGACCCGCGTCGCATTAGCTAGTT
GGTGAGGTAACGGCTCACCAAGGCAACGATGCGTAGCCGACCTGAGAGGGTGATCGGCCACACTGGGACTGAGA
CACGGCCCAGACTCCTACGGGAGGCAGCAGTAGGGAATCTTCCGCAATGGACGAAAGTCTGACGGAGCAACGCC
GCGTGAGTGATGAAGGCTTTCGGGTCGTAAAACTCTGTTGTTAGGGAAGAACAAGTGCTAGTTGAATAAGCTGGC
ACCTTGACGGTACCTAACCAGAAAGCCACGGCTAACTACGTGCCAGCAGCCGCGGTAATACGTAGGTGGCAAGCG
TTATCCGGAATTATTGGGCGTAAAGCGCGCGCAGGTGGTTTCTTAAGTCTGATGTGAAAGCCCACGGCTCAACCG
TGGAGGGTCATTGGAAACTGGGAGACTTGAGTGCAGAAGAGGAAAGTGGAATTCCATGTGTAGCGGTGAAATGC
GTAGAGATATGGAGGAACACCAGTGGCGAAGGCGACTTTCTGGTCTGTAACTGACACTGAGGCGCGAAAGCGTG
GGGAGCAAACAGGATTAGATACCCTGGTAGTCCACGCCGTAAACGATGAGTGCTAAGTGTTAGAGGGTTTCCGCC
CTTTAGTGCTGAAGTTAACGCATTAAGCACTCCGCCTGGGGAGTACGGCCGCAAGGCTGAAACTCAAAGGAATTG
ACGGGGGCCCGCACAAGCGGTGGAGCATGTGGTTTAATTCGAAGCAACGCGAAGAACCTTACCAGGTCTTGACAT
CCTCTGAAAACCCTAGAGATAGGGCTTCTCCTTCGGGAGCAGAGTGACAGGTGGTGCATGGTTGTCGTCAGCTCG
TGTCGTGAGATGTTGGGTTAAGTCCCGCAACGAGCGCAACCCTTGATCTTAGTTGCCATCATTAAGTTGGGCACTC
TAAGGTGACTGCCGGTGACAAACCGGAGGAAGGTGGGGATGACGTCAAATCATCATGCCCCTTATGACCTGGGCT
ACACACGTGCTACAATGGACGGTACAAAGAGCTGCAAGACCGCGAGGTGGAGCTAATCTCATAAAACCGTTCTCA
GTTCGGATTGTAGGCTGCAACTCGCCTACATGAAGCTGGAATCGCTAGTAATCGCGGATCAGCATGCCGCGGTGA
ATACGTTCCCGGGCCTTGTACACACCGCCCGTCACACCACGAGAGTTTGTAACACCCGAAGTCGGTGGGGTAACCT
TTATGGAGCCAGCCGCCTAAGGTGGGACAGATGATTGGGGTGAAGTCGTAGCAAGTACCCTGAGGG

3. ***Curtobacterium* sp., MZ424740** (s1860-3,9,15,21), 1514 bp.
AGAGTTTGATCCTGGCTCAGGACGAACGCTGGCGGCGTGCTTAACACATGCAAGTCGAACGATGATGCCCAGCTT
GCTGGGTGGATTAGTGGCGAACGGGTGAGTAACACGTGAGTAACCTGCCCCTGACTCTGGGATAAGCGTTGGAA
ACGACGTCTAATACTGGATATGATCACTGGCCGCATGGTCTGGTGGTGGAAAGATTTTTTGGTTGGGGATGGACT
CGCGGCCTATCAGCTTGTTGGTGAGGTAATGGCTCACCAAGGCGACGACGGGTAGCCGGCCTGAGAGGGTGACC
GGCCACACTGGGACTGAGACACGGCCCAGACTCCTACGGGAGGCAGCAGTGGGGAATATTGCACAATGGGCGAA
AGCCTGATGCAGCAACGCCGCGTGAGGGATGACGGCCTTCGGGTTGTAAACCTCTTTTAGTAGGGAAGAAGCGA
AAGTGACGGTACCTGCAGAAAAAGCACCGGCTAACTACGTGCCAGCAGCCGCGGTAATACGTAGGGTGCAAGCG
TTGTCCGGAATTATTGGGCGTAAAGAGCTCGTAGGCGGTTTGTCGCGTCTGCTGTGAAATCCCGAGGCTCAACCTC
GGGCTTGCAGTGGGTACGGGCAGACTAGAGTGCGGTAGGGGAGATTGGAATTCCTGGTGTAGCGGTGGAATGC
GCAGATATCAGGAGGAACACCGATGGCGAAGGCAGATCTCTGGGCCGTAACTGACGCTGAGGAGCGAAAGCAT

GGGGAGCGAACAGGATTAGATACCCTGGTAGTCCATGCCGTAAACGTTGGGCGCTAGATGTAGGGACCTTTCCAC
GGTTTCTGTGTCGTAGCTAACGCATTAAGCGCCCCGCCTGGGGAGTACGGCCGCAAGGCTAAAACTCAAAGGAAT
TGACGGGGGCCCGCACAAGCGGCGGAGCATGCGGATTAATTCGATGCAACGCGAAGAACCTTACCAAGGCTTGA
CATACACCGGAAACGGCCAGAGATGGTCGCCCCCTTGTGGTCGGTGTACAGGTGGTGCATGGTTGTCGTCAGCTC
GTGTCGTGAGATGTTGGGTTAAGTCCCGCAACGAGCGCAACCCTCGTTCTATGTTGCCAGCGGGTTATGCCGGGG
ACTCATAGGAGACTGCCGGGGTCAACTCGGAGGAAGGTGGGGATGACGTCAAATCATCATGCCCCTTATGTCTTG
GGCTTCACGCATGCTACAATGGCCGGTACAAAGGGCTGCGATACCGTAAGGTGGAGCGAATCCCAAAAAGCCGG
TCTCAGTTCGGATTGAGGTCTGCAACTCGACCTCATGAAGTCGGAGTCGCTAGTAATCGCAGATCAGCAACGCTGC
GGTGAATACGTTCCCGGGCCTTGTACACACCGCCCGTCAAGTCATGAAAGTCGGTAACACCCGAAGCCGGTGGCC
TAACCCTTGTGGAAGGAGCCGTCGAAGGTGGGATCGGTGATTAGGACTAAGTCGTAACAAGGTAGCCGTACCGG
AAGGTGCGGCTGGATCACCTCCTT

Curtobacterium flaccumfaciens, AJ310414.1, 1514 bp., Percent identity 100%
AGAGTTTGATCCTGGCTCAGGACGAACGCTGGCGGCGTGCTTAACACATGCAAGTCGAACGATGATGCCCAGCTT
GCTGGGTGGATTAGTGGCGAACGGGTGAGTAACACGTGAGTAACCTGCCCCTGACTCTGGGATAAGCGTTGGAA
ACGACGTCTAATACTGGATATGATCACTGGCCGCATGGTCTGGTGGTGGAAAGATTTTTTGGTTGGGGATGGACT
CGCGGCCTATCAGCTTGTTGGTGAGGTAATGGCTCACCAAGGCGACGACGGGTAGCCGGCCTGAGAGGGTGACC
GGCCACACTGGGACTGAGACACGGCCCAGACTCCTACGGGAGGCAGCAGTGGGGAATATTGCACAATGGGCGAA
AGCCTGATGCAGCAACGCCGCGTGAGGGATGACGGCCTTCGGGTTGTAAACCTCTTTTAGTAGGGAAGAAGCGA
AAGTGACGGTACCTGCAGAAAAAGCACCGGCTAACTACGTGCCAGCAGCCGCGGTAATACGTAGGGTGCAAGCG
TTGTCCGGAATTATTGGGCGTAAAGAGCTCGTAGGCGGTTTGTCGCGTCTGCTGTGAAATCCCGAGGCTCAACCTC
GGGCTTGCAGTGGGTACGGGCAGACTAGAGTGCGGTAGGGGAGATTGGAATTCCTGGTGTAGCGGTGGAATGC
GCAGATATCAGGAGGAACACCGATGGCGAAGGCAGATCTCTGGGCCGTAACTGACGCTGAGGAGCGAAAGCAT
GGGGAGCGAACAGGATTAGATACCCTGGTAGTCCATGCCGTAAACGTTGGGCGCTAGATGTAGGGACCTTTCCAC
GGTTTCTGTGTCGTAGCTAACGCATTAAGCGCCCCGCCTGGGGAGTACGGCCGCAAGGCTAAAACTCAAAGGAAT
TGACGGGGGCCCGCACAAGCGGCGGAGCATGCGGATTAATTCGATGCAACGCGAAGAACCTTACCAAGGCTTGA
CATACACCGGAAACGGCCAGAGATGGTCGCCCCCTTGTGGTCGGTGTACAGGTGGTGCATGGTTGTCGTCAGCTC
GTGTCGTGAGATGTTGGGTTAAGTCCCGCAACGAGCGCAACCCTCGTTCTATGTTGCCAGCGGGTTATGCCGGGG
ACTCATAGGAGACTGCCGGGGTCAACTCGGAGGAAGGTGGGGATGACGTCAAATCATCATGCCCCTTATGTCTTG
GGCTTCACGCATGCTACAATGGCCGGTACAAAGGGCTGCGATACCGTAAGGTGGAGCGAATCCCAAAAAGCCGG
TCTCAGTTCGGATTGAGGTCTGCAACTCGACCTCATGAAGTCGGAGTCGCTAGTAATCGCAGATCAGCAACGCTGC
GGTGAATACGTTCCCGGGCCTTGTACACACCGCCCGTCAAGTCATGAAAGTCGGTAACACCCGAAGCCGGTGGCC
TAACCCTTGTGGAAGGAGCCGTCGAAGGTGGGATCGGTGATTAGGACTAAGTCGTAACAAGGTAGCCGTACCGG
AAGGTGCGGCTGGATCACCTCCTT

4. ***Erwinia* sp., MZ424741** (s1860-4,10,16,22), 1508 bp.
AAGAGTTTGATCATGGCTCAGATTGAACGCTGGCGGCAGGCCTAACACATGCAAGTCGAACGGTAGCACAGAGA
GCTTGCTCTCGGGTGACGAGTGGCGGACGGGTGAGTAATGTCTGGGAAACTGCCTGATGGAGGGGGATAACTAC
TGGAAACGGTAGCTAATACCGCATAACGTCTTCGGACCAAAGTGGGGGACCTTCGGGCCTCACACCATCGGATGT
GCCCAGATGGGATTAGCTAGTAGGTGGGGTAATGGCTCACCTAGGCGACGATCCCTAGCTGGTCTGAGAGGATG
ACCAGCCACACTGGAACTGAGACACGGTCCAGACTCCTACGGGAGGCAGCAGTGGGGAATATTGCACAATGGGC
GCAAGCCTGATGCAGCCATGCCGCGTGTATGAAGAAGGCCTTCGGGTTGTAAAGTACTTTCAGCGGGGAGGAAG
GCGATAAGGTTAATAACCTTGTCGATTGACGTTACCCGCAGAAGAAGCACCGGCTAACTCCGTGCCAGCAGCCGC
GGTAATACGGAGGGTGCAAGCGTTAATCGGAATTACTGGGCGTAAAGCGCACGCAGGCGGTCTGTCAAGTCAGA
TGTGAAATCCCCGGGCTTAACCTGGGAACTGCATTTGAAACTGGCAGGCTAGAGTCTTGTAGAGGGGGGTAGAAT
TCCAGGTGTAGCGGTGAAATGCGTAGAGATCTGGAGGAATACCGGTGGCGAAGGCGGCCCCCTGGACAAAGACT
GACGCTCAGGTGCGAAAGCGTGGGGAGCAAACAGGATTAGATACCCTGGTAGTCCACGCCGTAAACGATGTCGA
CTTGGAGGTTGTGCCCTTGAGGCGTGGCTTCCGGAGCTAACGCGTTAAGTCGACCGCCTGGGGAGTACGGCCGCA
AGGTTAAAACTCAAATGAATTGACGGGGGCCCGCACAAGCGGTGGAGCATGTGGTTTAATTCGATGCAACGCGA
AGAACCTTACCTGGCCTTGACATCCACGGAATTCGGCAGAGATGCCTTAGTGCCTTCGGGAACCGTGAGACAGGT
GCTGCATGGCTGTCGTCAGCTCGTGTTGTGAAATGTTGGGTTAAGTCCCGCAACGAGCGCAACCCTTATCCTTTGT
TGCCAGCGAGTAATGTCGGGAACTCAAAGGAGACTGCCGGTGACAAACCGGAGGAAGGTGGGGATGACGTCAA
GTCATCATGGCCCTTACGGCCAGGGCTACACACGTGCTACAATGGCGCATACAAAGAGAAGCGAACTCGCGAGA
GCAAGCGGACCTCACAAAGTGCGTCGTAGTCCGGATCGGAGTCTGCAACTCGACTCCGTGAAGTCGGAATCGCTA

GTAATCGTAGATCAGAATGCTACGGTGAATACGTTCCCGGGCCTTGTACACACCGCCCGTCACACCATGGGAGTG
GGTTGCAAAAGAAGTAGGTAGCTTAACCTTCGGGAGGGCGCTTACCACTTTGTGATTCATGACTGGGGTGAAGTC
GTAACAAGGTAACCGTAGG

Erwinia billingiae, KM408608.1, 1535 bp., Percent identity 100%
GAAGAGTTTGATCATGGCTCAGATTGAACGCTGGCGGCAGGCCTAACACATGCAAGTCGAACGGTAGCACAGAG
AGCTTGCTCTCGGGTGACGAGTGGCGGACGGGTGAGTAATGTCTGGGAAACTGCCTGATGGAGGGGGATAACTA
CTGGAAACGGTAGCTAATACCGCATAACGTCTTCGGACCAAAGTGGGGGACCTTCGGGCCTCACACCATCGGATG
TGCCCAGATGGGATTAGCTAGTAGGTGGGGTAATGGCTCACCTAGGCGACGATCCCTAGCTGGTCTGAGAGGAT
GACCAGCCACACTGGAACTGAGACACGGTCCAGACTCCTACGGGAGGCAGCAGTGGGGAATATTGCACAATGGG
CGCAAGCCTGATGCAGCCATGCCGCGTGTATGAAGAAGGCCTTCGGGTTGTAAAGTACTTTCAGCGGGGAGGAA
GGCGATAAGGTTAATAACCTTGTCGATTGACGTTACCCGCAGAAGAAGCACCGGCTAACTCCGTGCCAGCAGCCG
CGGTAATACGGAGGGTGCAAGCGTTAATCGGAATTACTGGGCGTAAAGCGCACGCAGGCGGTCTGTCAAGTCAG
ATGTGAAATCCCCGGGCTTAACCTGGGAACTGCATTTGAAACTGGCAGGCTAGAGTCTTGTAGAGGGGGGTAGA
ATTCCAGGTGTAGCGGTGAAATGCGTAGAGATCTGGAGGAATACCGGTGGCGAAGGCGGCCCCCTGGACAAAGA
CTGACGCTCAGGTGCGAAAGCGTGGGGAGCAAACAGGATTAGATACCCTGGTAGTCCACGCCGTAAACGATGTC
GACTTGGAGGTTGTGCCCTTGAGGCGTGGCTTCCGGAGCTAACGCGTTAAGTCGACCGCCTGGGGAGTACGGCC
GCAAGGTTAAAACTCAAATGAATTGACGGGGGCCCGCACAAGCGGTGGAGCATGTGGTTTAATTCGATGCAACG
CGAAGAACCTTACCTGGCCTTGACATCCACGGAATTCGGCAGAGATGCCTTAGTGCCTTCGGGAACCGTGAGACA
GGTGCTGCATGGCTGTCGTCAGCTCGTGTTGTGAAATGTTGGGTTAAGTCCCGCAACGAGCGCAACCCTTATCCTT
TGTTGCCAGCGAGTAATGTCGGGAACTCAAAGGAGACTGCCGGTGACAAACCGGAGGAAGGTGGGGATGACGT
CAAGTCATCATGGCCCTTACGGCCAGGGCTACACACGTGCTACAATGGCGCATACAAAGAGAAGCGAACTCGCGA
GAGCAAGCGGACCTCACAAAGTGCGTCGTAGTCCGGATCGGAGTCTGCAACTCGACTCCGTGAAGTCGGAATCGC
TAGTAATCGTAGATCAGAATGCTACGGTGAATACGTTCCCGGGCCTTGTACACACCGCCCGTCACACCATGGGAGT
GGGTTGCAAAAGAAGTAGGTAGCTTAACCTTCGGGAGGGCGCTTACCACTTTGTGATTCATGACTGGGGTGAAGT
CGTAACAAGGTAACCGTAGGGGAACCTGCGGTTGGATCACCTCCTT

5. ***Pantoea* sp., MZ424742** (s1706-9-al14), 419 bp.
GCTGGCAGGCCTAACACATGCAAGTCGGACGGTAGCACAGAGGAGCTTGCTCCTTGGTGGACGAGTGCCGAACG
GGTAAGTAATGTCGGGGAACCGCCCCGTAAAAGGGGGATAACCACTGGAAACGGTGGCTAATACCGCATAACGT
CGCAAGACAAAAGAGGGGACCTTCGGGCCTCTCACTATCGGATGAAACCAGATGGGATTAGCTAGTAGGCGGGG
TAATGGCCCAACTAGGCGACCAACCCTAACCGGGCTGAAAGGAAGGACCAGCAACACGGAAATGAGAAAACGTC
CAGAATCCCACCGGGAGGCACCAGTGGGGAAAATTGCACAATGGGCGCAAGCCTGATGCAGCCATGCCGCGTGT
ATGAAGAAGGCCTTCGGGTTGTAAAGTACTTTCAGCGGGGAGGAAGGCG

Pantoea agglomerans, MT605813.1, 1505bp., Percent identity 99.75%
AGAGTTTGATCATGGCTCAGATTGAACGCTGGCGGCAGGCCTAACACATGCAAGTCGGACGGTAGCACAGAGGA
GCTTGCTCCTTGGGTGACGAGTGGCGGACGGGTGAGTAATGTCTGGGGATCTGCCCGATAGAGGGGGATAACCA
CTGGAAACGGTGGCTAATACCGCATAACGTCGCAAGACCAAAGAGGGGGACCTTCGGGCCTCTCACTATCGGATG
AACCCAGATGGGATTAGCTAGTAGGCGGGGTAATGGCCCACCTAGGCGACGATCCCTAGCTGGTCTGAGAGGAT
GACCAGCCACACTGGAACTGAGACACGGTCCAGACTCCTACGGGAGGCAGCAGTGGGGAATATTGCACAATGGG
CGCAAGCCTGATGCAGCCATGCCGCGTGTATGAAGAAGGCCTTCGGGTTGTAAAGTACTTTCAGCGGGGAGGAA
GGCGACGGGGTTAATAACCCTGTCGATTGACGTTACCCGCAGAAGAAGCACCGGCTAACTCCGTGCCAGCAGCCG
CGGTAATACGGAGGGTGCAAGCGTTAATCGGAATTACTGGGCGTAAAGCGCACGCAGGCGGTCTGTTAAGTCAG
ATGTGAAATCCCCGGGCTTAACCTGGGAACTGCATTTGAAACTGGCAGGCTTGAGTCTTGTAGAGGGGGGTAGAA
TTCCAGGTGTAGCGGTGAAATGCGTAGAGATCTGGAGGAATACCGGTGGCGAAGGCGGCCCCCTGGACAAAGAC
TGACGCTCAGGTGCGAAAGCGTGGGGAGCAAACAGGATTAGATACCCTGGTAGTCCACGCCGTAAACGATGTCG
ACTTGGAGGTTGTTCCCTTGAGGAGTGGCTTCCGGAGCTAACGCGTTAAGTCGACCGCCTGGGGAGTACGGCCGC
AAGGTTAAAACTCAAATGAATTGACGGGGGCCCGCACAAGCGGTGGAGCATGTGGTTTAATTCGATGCAACGCG
AAGAACCTTACCTACTCTTGACATCCACGGAATTTGGCAGAGATGCCTTAGTGCCTTCGGGAACCGTGAGACAGGT
GCTGCATGGCTGTCGTCAGCTCGTGTTGTGAAATGTTGGGTTAAGTCCCGCAACGAGCGCAACCCTTATCCTTTGT
TGCCAGCGATTCGGTCGGGAACTCAAAGGAGACTGCCGGTGATAAACCGGAGGAAGGTGGGGATGACGTCAAG
TCATCATGGCCCTTACGAGTAGGGCTACACACGTGCTACAATGGCGCATACAAAGAGAAGCGACCTCGCGAGAGC
AAGCGGACCTCACAAAGTGCGTCGTAGTCCGGATCGGAGTCTGCAACTCGACTCCGTGAAGTCGGAATCGCTAGT

AATCGTGGATCAGAATGCCACGGTGAATACGTTCCCGGGCCTTGTACACACCGCCCGTCACACCATGGGAGTGGG
TTGCAAAAGAAGTAGGTAGCTTAACCTTCGGGAGGGCGCTTACCACTTTGTGATTCATGACTGGGGTGAAGTCGT
AACAAGGTACCATC

6. ***Pseudomonas* sp., MZ424743** (s1860-5,11,17,23), 1530 bp.
AAGAGTTTGATCATGGCTCAGATTGACCGCTGGCGGCAGGCCTAACACATGCAAGTCGAGCGGATGAGAGGAGC
TTGCTCTTCGATTCAGCGGCGGACGGGTGAGTAATGCCTAGGAATCTGCCTGGTAGTGGGGGACAACGTTTCGAA
AGGAACGCTAATACCGCATACGTCCTACGGGAGAAAGCAGGGGACCTTCGGGCCTTGCGCTATCAGATGAGCCTA
GGTCGGATTAGCTAGTTGGTGAGGTAATGGCTCACCAAGGCGACGATCCGTAACTGGTCTGAGAGGATGATCAGT
CACACTGGAACTGAGACACGGTCCAGACTCCTACGGGAGGCAGCAGTGGGGAATATTGGACAATGGGCGAAAGC
CTGATCCAGCCATGCCGCGTGTGTGAAGAAGGTCTTCGGATTGTAAAGCACTTTAAGTTGGGAGGAAGGGTTGTT
TCCTAATACGAAGCAATTTTGACGTTACCGACAGAATAAGCACCGGCTAACTCTGTGCCAGCAGCCGCGGTAATAC
AGAGGGTGCAAGCGTTAATCGGAATTACTGGGCGTAAAGCGCGCGTAGGTGGTTTGTTAAGTTGAATGTGAAATC
CCCGGGCTCAACCTGGGAACTGCATCCAAAACTGGCAAGCTAGAGTAGGGCAGAGGGTGGTGGAATTTCCTGTG
TAGCGGTGAAATGCGTAGATATAGGAAGGAACACCAGTGGCGAAGGCGACCACCTGGGCTCATACTGACACTGA
GGTGCGAAAGCGTGGGGAGCAAACAGGATTAGATACCCTGGTAGTCCACGCCGTAAACGATGTCAACTAGCCGT
TGGAATCCTTGAGATTTTAGTGGCGCAGCTAACGCATTAAGTTGACCGCCTGGGGAGTACGGCCGCAAGGTTAAA
ACTCAAATGAATTGACGGGGGCCCGCACAAGCGGTGGAGCATGTGGTTTAATTCGAAGCAACGCGAAGAACCTT
ACCAGGCCTTGACATCCAATGAACTTTCCAGAGATGGATTGGTGCCTTCGGGAACATTGAGACAGGTGCTGCATG
GCTGTCGTCAGCTCGTGTCGTGAGATGTTGGGTTAAGTCCCGTAACGAGCGCAACCCTTGTCCTTAGTTACCAGCA
CGTTATGGTGGGCACTCTAAGGAGACTGCCGGTGACAAACCGGAGGAAGGTGGGGATGACGTCAAGTCATCATG
GCCCTTACGGCCTGGGCTACACACGTGCTACAATGGTCGGTACAGAGGGTTGCCAAGCCGCGAGGTGGAGCTAAT
CTCACAAAACCGATCGTAGTCCGGATCGCAGTCTGCAACTCGACTGCGTGAAGTCGGAATCGCTAGTAATCGCGA
ATCAGAATGTCGCGGTGAATACGTTCCCGGGCCTTGTACACACCGCCCGTCACACCATGGGAGTGGGTTGCACCA
GAAGTAGCTAGTCTAACCTTCGGGAGGACGGTTACCACGGTGTGATTCATGACTGGGGTGAAGTCGTAACAAGGT
AGCCGTAGGGGAACCTGCGGCTGGATCACCTCCTT

Pseudomonas alkylphenolica, MN813762.1, 1542 bp., Percent identity
98.89%ATTGAACTGAAGAGTTTGATCATGGCTCAGATTGAACGCTGGCGGCAGGCCTAACACATGCAAGTCGAGC
GGATGAGAAGAGCTTGCTCTTCGATTCAGCGGCGGACGGGTGAGTAATACCTAGGAATCTGCCTGGTAGTGGGG
GACAACGTTTCGAAAGGAACGCTAATACCGCATACGTCCTACGGGAGAAAGCAGGGGACCTTCGGGCCTTGCGCT
ATCAGATGAGCCTAGGTCGGATTAGCTAGTTGGTGAGGTAATGGCTCACCAAGGCGACGATCCGTAACTGGTCTG
AGAGGATGATCAGTCACACTGGAACTGAGACACGGTCCAGACTCCTACGGGAGGCAGCAGTGGGGAATATTGGA
CAATGGGCGAAAGCCTGATCCAGCCATGCCGCGTGTGTGAAGAAGGTCTTCGGATTGTAAAGCACTTTAAGTTGG
GAGGAAGGGTACTTACCTAATACGTGAGTATTTTGACGTTACCGACAGAATAAGCACCGGCTAACTCTGTGCCAG
CAGCCGCGGTAATACAGAGGGTGCAAGCGTTAATCGGAATTACTGGGCGTAAAGCGCGCGTAGGTGGTTTGTTA
AGTTGGATGTGAAATCCCCGGGCTCAACCTGGGAACTGCATCCAAAACTGGCAAGCTAGAGTAGGGCAGAGGGT
GGTGGAATTTCCTGTGTAGCGGTGAAATGCGTAGATATAGGAAGGAACACCAGTGGCGAAGGCGACCACCTGGG
CTCATACTGACACTGAGGTGCGAAAGCGTGGGGAGCAAACAGGATTAGATACCCTGGTAGTCCACGCCGTAAAC
GATGTCAACTAGCCGTTGGAATCCTTGAGATTTTAGTGGCGCAGCTAACGCATTAAGTTGACCGCCTGGGGAGTA
CGGCCGCAAGGTTAAAACTCAAATGAATTGACGGGGGCCCGCACAAGCGGTGGAGCATGTGGTTTAATTCGAAG
CAACGCGAAGAACCTTACCAGGCCTTGACATGCAGAGAACTTTCCAGAGATGGATTGGTGCCTTCGGGAACTCTG
ACACAGGTGCTGCATGGCTGTCGTCAGCTCGTGTCGTGAGATGTTGGGTTAAGTCCCGTAACGAGCGCAACCCTT
GTCCTTAGTTACCAGCACGTTATGGTGGGCACTCTAAGGAGACTGCCGGTGACAAACCGGAGGAAGGTGGGGAT
GACGTCAAGTCATCATGGCCCTTACGGCCTGGGCTACACACGTGCTACAATGGTCGGTACAGAGGGTTGCCAAGC
CGCGAGGTGGAGCTAATCTCACAAAACCGATCGTAGTCCGGATCGCAGTCTGCAACTCGACTGCGTGAAGTCGGA
ATCGCTAGTAATCGCGAATCAGAATGTCGCGGTGAATACGTTCCCGGGCCTTGTACACACCGCCCGTCACACCATG
GGAGTGGGTTGCACCAGAAGTAGCTAGTCTAACCTTCGGGAGGACGGTTACCACGGTGTGATTCATGACTGGGG
TGAAGTCGTAACAAGGTAGCCGTAGGGGAACCTGCGGCTGGATCACCTCCTTAAT

7. ***Xanthomonas* sp., MZ424744** (s1860-6,12,18,24), 1497
bp.AGTGAACGCTGGCGGCAGGCCTAACACATGCAAGTCGAACGGCAGCACAGAGGAGCTTGCTCCTTGGGTGGC
GAGTGGCGGACGGGTGAGGAATACATCGGAATCTACTCTGTCGTGGGGGATAACGTAGGGAAACTTACGCTAAT
ACCGCATACGACCTACGGGTGAAAGCGGAGGACCTTCGGGCTTCGCGCGATTGAATGAGCCGATGTCGGATTAG

CTAGTTGGCGGGGTAAAGGCCCACCAAGGCGACGATCCGTAGCTGGTCTGAGAGGATGATCAGCCACACTGGAA
CTGAGACACGGTCCAGACTCCTACGGGAGGCAGCAGTGGGGAATATTGGACAATGGGCGCAAGCCTGATCCAGC
CATGCCGCGTGGGTGAAGAAGGCCTTCGGGTTGTAAAGCCCTTTTGTTGGGAAAGAAAAGCAGTCGGTTAATACC
CGATTGTTCTGACGGTACCCAAAGAATAAGCACCGGCTAACTTCGTGCCAGCAGCCGCGGTAATACGAAGGGTGC
AAGCGTTACTCGGAATTACTGGGCGTAAAGCGTGCGTAGGTGGTGGTTTAAGTCCGTTGTGAAAGCCCTGGGCTC
AACCTGGGAATTGCAGTGGATACTGGGTCACTAGAGTGTGGTAGAGGGTAGCGGAATTCCCGGTGTAGCAGTGA
AATGCGTAGAGATCGGGAGGAACATCTGTGGCGAAGGCGGCTACCTGGACCAACACTGACACTGAGGCACGAAA
GCGTGGGGAGCAAACAGGATTAGATACCCTGGTAGTCCACGCCCTAAACGATGCGAACTGGATGTTGGGTGCAA
TTTGGCACGCAGTATCGAAGCTAACGCGTTAAGTTCGCCGCCTGGGGAGTACGGTCGCAAGACTGAAACTCAAAG
GAATTGACGGGGGCCCGCACAAGCGGTGGAGTATGTGGTTTAATTCGATGCAACGCGAAGAACCTTACCTGGTCT
TGACATCCACGGAACTTTCCAGAGATGGATTGGTGCCTTCGGGAACCGTGAGACAGGTGCTGCATGGCTGTCGTC
AGCTCGTGTCGTGAGATGTTGGGTTAAGTCCCGCAACGAGCGCAACCCTTGTCCTTAGTTGCCAGCACGTAATGGT
GGGAACTCTAAGGAGACCGCCGGTGACAAACCGGAGGAAGGTGGGGATGACGTCAAGTCATCATGGCCCTTACG
ACCAGGGCTACACACGTACTACAATGGTTAGGACAGAGGGCTGCAATCCCGCGAGGGTGAGCCAATCCCAGAAA
CCTAATCTCAGTCCGGATTGGAGTCTGCAACTCGACTCCATGAAGTCGGAATCGCTAGTAATCGCAGATCAGCATT
GCTGCGGTGAATACGTTCCCGGGCCTTGTACACACCGCCCGTCACACCATGGGAGTTTGTTGCACCAGAAGCAGG
TAGCTTAACCTTCGGGAGGGCGCTTGCCACGGTGTGGCCGATGACTGGGGTGAAGTCGTAACAAGGTAGCCGTA
TCGGAAGG

Xanthomonas campestris, MN108237,1547 bp., Percent identity
99.13%TAAGTGAAGAGTTTGATCCTGGCTCAGAGTGAACGCTGGCGGCAGGCCTAACACATGCAAGTCGAACGG
CAGCACAGTAAGAGCTTGCTCTTATGGGTGGCGAGTGGCGGACGGGTGAGGAATACATCGGAATCTACTCTTTCG
TGGGGGATAACGTAGGGAAACTTACGCTAATACCGCATACGACCTACGGGTGAAAGCGGAGGACCTTCGGGCTT
CGCGCGATTGAATGAGCCGATGTCGGATTAGCTAGTTGGCGGGGTAAAGGCCCACCAAGGCGACGATCCGTAGC
TGGTCTGAGAGGATGATCAGCCACACTGGAACTGAGACACGGTCCAGACTCCTACGGGAGGCAGCAGTGGGGAA
TATTGGACAATGGGCGCAAGCCTGATCCAGCCATGCCGCGTGGGTGAAGAAGGCCTTCGGGTTGTAAAGCCCTTT
TGTTGGGAAAGAAAAGCAGTCGGTTAATACCCGATTGTTCTGACGGTACCCAAAGAATAAGCACCGGCTAACTTC
GTGCCAGCAGCCGCGGTAATACGAAGGGTGCAAGCGTTACTCGGAATTACTGGGCGTAAAGCGTGCGTAGGTGG
TGGTTTAAGTCTGTTGTGAAAGCCCTGGGCTCAACCTGGGAATTGCAGTGGATACTGGGTCACTAGAGTGTGGTA
GAGGGTAGCGGAATTCCCGGTGTAGCAGTGAAATGCGTAGAGATCGGGAGGAACATCCGTGGCGAAGGCGGCT
ACCTGGACCAACACTGACACTGAGGCACGAAAGCGTGGGGAGCAAACAGGATTAGATACCCTGGTAGTCCACGC
CCTAAACGATGCGAACTGGATGTTGGGTGCAATTTGGCACGCAGTATCGAAGCTAACGCGTTAAGTTCGCCGCCT
GGGGAGTACGGTCGCAAGACTGAAACTCAAAGGAATTGACGGGGGCCCGCACAAGCGGTGGAGTATGTGGTTTA
ATTCGATGCAACGCGAAGAACCTTACCTGGTCTTGACATCCACGGAACTTTCCAGAGATGGATTGGTGCCTTCGGG
AACCGTGAGACAGGTGCTGCATGGCTGTCGTCAGCTCGTGTCGTGAGATGTTGGGTTAAGTCCCGCAACGAGCGC
AACCCTTGTCCTTAGTTGCCAGCACGTAATGGTGGGAACTCTAAGGAGACCGCCGGTGACAAACCGGAGGAAGGT
GGGGATGACGTCAAGTCATCATGGCCCTTACGACCAGGGCTACACACGTACTACAATGGTAGGGACAGAGGGCT
GCAAACCCGCGAGGGTAAGCCAATCCCAGAAACCCTATCTCAGTCCGGATTGGAGTCTGCAACTCGACTCCATGA
AGTCGGAATCGCTAGTAATCGCAGATCAGCATTGCTGCGGTGAATACGTTCCCGGGCCTTGTACACACCGCCCGTC
ACACCATGGGAGTTTGTTGCACCAGAAGCAGGTAGCTTAACCTTCGGGAGGGCGCTTGCCACGGTGTGGCCGATG
ACTGGGGTGAAGTCGTAACAAGGTAGCCGTATCGGAAGGTGCGGCTGGATCACCTCCTTT

8. ***Alternaria* sp., MZ427922** (s1778-2-al14), 562 bp.
TCCGTAGGTGAACCTGCGGAGGGATCATTACACAAATATGAAGGCGGGCTGGAACCTCTCGGGGTTACAGCCTTG
CTGAATTATTCACCCTTGTCTTTTGCGTACTTCTTGTTTCCTTGGTGGGTTCGCCCACCACTAGGACAAACATAAACC
TTTTGTAATTGCAATCAGCGTCAGTAACAAATTAATAATTACAACTTTCAACAACGGATCTCTTGGTTCTGGCATCG
ATGAAGAACGCAGCGAAATGCGATAAGTAGTGTGAATCGCAGAATTCAGTGAATCATCGAATCTTTGAACGCACA
TTGCGCCCTTTGGTATTCCAAAGGGCATGCCTGTTCGAGCGTCATTTGTACCCTCAAGCTTTGCTTGGTGTTGGGCG
TCTTGTCTCTAGCTTTGCTGGAGACTCGCCTTAAAGTAATTGGCAGCCGGCCTACTGGTTTCGGAGCGCAGCACAA
GTCGCACTCTCTATCAGCAAAGGTCTAGCATCCATTAAGCCTTTTTTTCAACTTTTGACCTCGGATCAGGTAGGGAT
ACCCGCTGAACTTAAGCATATCAATAA

Alternaria tenuissima, KF308883.1, 570bp., Percent identity 100%

TCCGTAGGTGAACCTGCGGAGGGATCATTACACAAATATGAAGGCGGGCTGGAACCTCTCGGGGTTACAGCCTTG
CTGAATTATTCACCCTTGTCTTTTGCGTACTTCTTGTTTCCTTGGTGGGTTCGCCCACCACTAGGACAAACATAAACC
TTTTGTAATTGCAATCAGCGTCAGTAACAAATTAATAATTACAACTTTCAACAACGGATCTCTTGGTTCTGGCATCG
ATGAAGAACGCAGCGAAATGCGATAAGTAGTGTGAATCGCAGAATTCAGTGAATCATCGAATCTTTGAACGCACA
TTGCGCCCTTTGGTATTCCAAAGGGCATGCCTGTTCGAGCGTCATTTGTACCCTCAAGCTTTGCTTGGTGTTGGGCG
TCTTGTCTCTAGCTTTGCTGGAGACTCGCCTTAAAGTAATTGGCAGCCGGCCTACTGGTTTCGGAGCGCAGCACAA
GTCGCACTCTCTATCAGCAAAGGTCTAGCATCCATTAAGCCTTTTTTTCAACTTTTGACCTCGGATCAGGTAGGGAT
ACCCGCTGAACTTAAGCATATCAATAAGCGGAGGA

9. ***Biscogniauxia* sp., MZ427923** (s1778-10-al22), 590 bp
ATGTGAACATACCTACTGTTGCCTCGGCAGGTCGTGCTGCGCGGCAGGATCGCCCCCCCTCGGGTTCGGCTGCTAC
AGGACTAGCTACCCTGTAGCGGCTTTCCTGGAGTGGCTACCCTGGAGCAGCTGCTACTGGACTAGCTACCCCGTA
GCAGCTGCCCTGGGGCGGCTACCCCGCAGCGGGGGGGCGCCCGCCCCGCAGCACGCCAACAGGCCTGCCGGAG
GACCCCTAAACTCTGTTTTACACCTGTATCTCTGAGGCTATGATGGAAATAAGTTAAAACTTTCAACAACGGATCTC
TTGGCTCTGGCATCGATGAAGAACGCAGCGAAATGCGATAAGTAATGTGAATTGCAGAATTCAGTGAATCATCGA
ATCTTTGAACGCACATTGCGCCTGACAGTATTCTGTTAGGCAAGCCTGTTCGAGCGTCATTTCAACCCTCAAGCCCT
ATTTGCTTGACGTTGGGAGTTTACGGAGACGTAATTCCTTAAATATAGTGGCGGAGCCGGGTCGTGCTCTGGGCG
TAGTAACCAAACTCTCGCCTCTGTAGCCGGCTCGGGTCTTGCCGTAAAACCCCCTATATTCT

Biscogniauxia maritima, MN341558.1, 707 bp., Percent identity 100%
GTAACAAGGTCTCCGTTGGTGAACCAGCGGAGGGATCATTAGCGAGTTGGTTACAAGCTCCAAACCCATGTGAAC
ATACCTACTGTTGCCTCGGCAGGTCGTGCTGCGCGGCAGGATCGCCCCCCCTCGGGTTCGGCTGCTACAGGACTA
GCTACCCTGTAGCGGCTACCCTGGAGTGGCTACCCTGGAGCAGCTGCTACTGGACTAGCTACCCCGTAGCAGCTG
CCCTGGGGCAGCTACCCCGCAGCGGGGGGGCGCCCGCCCCGCAGCACGCCAACAGGCCTGCCGGAGGACCCCTA
AACTCTGTTTTACACCTGTATCTCTGAGGCTATGATGGAAATAAGTTAAAACTTTCAACAACGGATCTCTTGGCTCT
GGCATCGATGAAGAACGCAGCGAAATGCGATAAGTAATGTGAATTGCAGAATTCAGTGAATCATCGAATCTTTGA
ACGCACATTGCGCCTGACAGTATTCTGTTAGGCATGCCTGTTCGAGCGTCATTTCAACCCTCAAGCCCTATTTGCTT
GACGTTGGGAGTTTACGGAGACGTAATTCCTTAAATATAGTGGCGGAGCCGGGTCGTGCTCTGGGCGTAGTAACC
AAACTCTCGCCTCTGTAGCCGGCTCGGGTCTTGCCGTAAAACCCCCTATATTCTTCTGGTTGACCTCGGATCAGGTA
GGAATACCCGCTGAACTTAAGCATATC

10. ***Cladosporium sp,* MZ427924** (s1778-7-al19), 512 bp.
AGGGATCATTACAAGTGACCCCGGTCTTACCACCGGGATGTTCATAACCCTTTGTTGTCCGACTCTGTTGCCTCCGG
GGCGACCCTGCCTTCGGGCGGGGGCTCCGGGTGGACACTTCAAACTCTTGCGTAACTTTGCAGTCTGAGTAAACTT
AATTAATAAATTAAAACTTTTAACAACGGATCTCTTGGTTCTGGCATCGATGAAGAACGCAGCGAAATGCGATAAG
TAATGTGAATTGCAGAATTCAGTGAATCATCGAATCTTTGAACGCACATTGCGCCCCCTGGTATTCCGGGGGGCAT
GCCTGTTCGAGCGTCATTTCACCACTCAAGCCTCGCTTGGTATTGGGCAACGCGGTCCGCCGCGTGCCTCAAATCG
ACCGGCTGGGTCTTCTGTCCCCTAAGCGTTGTGGAAACTATTCGCTAAAGGGTGTTCGGGAGGCTACGCCGTAAA
ACAACCCCATTTCTAAGGTTGACCTCGGATCAGGTAGGGATACCCGCTGAACTTAA

Cladosporium perangustum, MT645918.1, 512 bp., Percent identity 100%
AGGGATCATTACAAGTGACCCCGGTCTTACCACCGGGATGTTCATAACCCTTTGTTGTCCGACTCTGTTGCCTCCGG
GGCGACCCTGCCTTCGGGCGGGGGCTCCGGGTGGACACTTCAAACTCTTGCGTAACTTTGCAGTCTGAGTAAACTT
AATTAATAAATTAAAACTTTTAACAACGGATCTCTTGGTTCTGGCATCGATGAAGAACGCAGCGAAATGCGATAAG
TAATGTGAATTGCAGAATTCAGTGAATCATCGAATCTTTGAACGCACATTGCGCCCCCTGGTATTCCGGGGGGCAT
GCCTGTTCGAGCGTCATTTCACCACTCAAGCCTCGCTTGGTATTGGGCAACGCGGTCCGCCGCGTGCCTCAAATCG
ACCGGCTGGGTCTTCTGTCCCCTAAGCGTTGTGGAAACTATTCGCTAAAGGGTGTTCGGGAGGCTACGCCGTAAA
ACAACCCCATTTCTAAGGTTGACCTCGGATCAGGTAGGGATACCCGCTGAACTTAA

11. ***Didymella* sp., MZ427925** (s1778-4-al16), 493 bp.
GAACCTGCGGAAGGATCATTACCTAGAGTTGTGGGCTTTGCCCGCCATCTCTTACCCATGTCTTTTGAGTACCTTCG
TTTCCTCGGCGGGTCCGCCCGCCGATTGGACAATTTAAACCATTTGCAGTTGCAATCAGCGTCTGAAAAAAACTTA
ATAGTTACAACTTTCAACAACGGATCTCTTGGTTCTGGCATCGATGAAGAACGCAGCGAAATGCGATAAGTAGTGT
GAATTGCAGAATTCAGTGAATCATCGAATCTTTGAACGCACATTGCGCCCCTTGGTATTCCATGGGGCATGCCTGT

TCGAGCGTCATTTGTACCTTCAAGCTCTGCTTGGTGTTGGGTGTTTGTCTCGCCTCCGCGTGTAGACTCGCCTCAAA
ACAATTGGCAGCCGGCGTATTGATTTCGGAGCGCAGTACATCTCGCGCTTTGCACTCATAACGACGACGTCCAAAA
GTACATTTTTTACACTCTTGAC

Didymella negriana, MK100201.1, 487 bp., Percent identity 100%
ATCATTACCTAGAGTTGTGGGCTTTGCCCGCCATCTCTTACCCATGTCTTTTGAGTACCTTCGTTTCCTCGGCGGGTC
CGCCCGCCGATTGGACAATTTAAACCATTTGCAGTTGCAATCAGCGTCTGAAAAAAACTTAATAGTTACAACTTTCA
ACAACGGATCTCTTGGTTCTGGCATCGATGAAGAACGCAGCGAAATGCGATAAGTAGTGTGAATTGCAGAATTCA
GTGAATCATCGAATCTTTGAACGCACATTGCGCCCCTTGGTATTCCATGGGGCATGCCTGTTCGAGCGTCATTTGTA
CCTTCAAGCTCTGCTTGGTGTTGGGTGTTTGTCTCGCCTCCGCGTGTAGACTCGCCTCAAAACAATTGGCAGCCGG
CGTATTGATTTCGGAGCGCAGTACATCTCGCGCTTTGCACTCATAACGACGACGTCCAAAAGTACATTTTTTACACT
CTTGACCTCGGATCAGGTAGGGATACC

12. ***Didymella* sp., MZ427926** (s1778-5-al17), 543 bp.
AGTCGTAACAAGGTTTCCGTAGGTGAACCTGCGGAAGGATCATTACCTAGAGTTGTGGGCTTTGCCCGCCATCTCT
TACCCATGTCTTTTGAGTACCTTCGTTTCCTCGGCGGGTCCGCCCGCCGATTGGACAATTTAAACCATTTGCAGTTG
CAATCAGCGTCTGAAAAAAACTTAATAGTTACAACTTTCAACAACGGATCTCTTGGTTCTGGCATCGATGAAGAAC
GCAGCGAAATGCGATAAGTAGTGTGAATTGCAGAATTCAGTGAATCATCGAATCTTTGAACGCACATTGCGCCCCT
TGGTATTCCATGGGGCATGCCTGTTCGAGCGTCATTTGTACCTTCAAGCTCTGCTTGGTGTTGGGTGTTTGTCTCGC
CTCCGCGTGTAGACTCGCCTCAAAACAATTGGCAGCCGGCGTATTGATTTCGGAGCGCAGTACATCTCGCGCTTTG
CACTCATAACGACGACGTCCAAAAGTACATTTTTTACACTCTTGACCTCGGATCAGGTAGGGATACCCGCTGAACTT
AAGCATAT

Didymella pinodella, KX869956.1, 957 bp., Percent identity 100%
TCTTGGTCCATTTAGAGGAAGTAAAAGTCGTAACAAGGTTTCCGTAGGTGAACCTGCGGAAGGATCATTACCTAG
AGTTGTGGGCTTTGCCCGCCATCTCTTACCCATGTCTTTTGAGTACCTTCGTTTCCTCGGCGGGTCCGCCCGCCGAT
TGGACAATTTAAACCATTTGCAGTTGCAATCAGCGTCTGAAAAAAACTTAATAGTTACAACTTTCAACAACGGATCT
CTTGGTTCTGGCATCGATGAAGAACGCAGCGAAATGCGATAAGTAGTGTGAATTGCAGAATTCAGTGAATCATCG
AATCTTTGAACGCACATTGCGCCCCTTGGTATTCCATGGGGCATGCCTGTTCGAGCGTCATTTGTACCTTCAAGCTC
TGCTTGGTGTTGGGTGTTTGTCTCGCCTCCGCGTGTAGACTCGCCTCAAAACAATTGGCAGCCGGCGTATTGATTT
CGGAGCGCAGTACATCTCGCGCTTTGCACTCATAACGACGACGTCCAAAAGTACATTTTTTACACTCTTGACCTCGG
ATCAGGTAGGGATACCCGCTGAACTTAAGCATATCAATAAGCGGAGGAAAAGAAACCAACAGGGATTGCCCTAGT
AACGGCGAGTGAAGCGGCAACAGCTCAAATTTGAAATCTGGCGTCTTTGGCGTCCGAGTTGTAATTTGCAGAGGG
CGCTTTGGCATTGGCAGCGGTCCAAGTTCCTTGGAACAGGACGTCACAGAGGGTGAGAATCCCGTACGTGGTCGC
TAGCCTTTACCGTGTAAAGCCCCTTCGACGAGTCGAGTTGTTTGGGAATGCAGCTCTAAATGGGAGGTAAATTTCT
TCTAAAGCTAAATACTGGCCAGAGACCGATAGCGCACAAGTAGAGTGATCGAAAGATGAAAAGCACTTTGGAAA
GAGAGTTAAAAAGCACGTGAAATTGTTGAAAGGAAAAAACACTTGAAA

13. ***Fusarium* sp., MZ427927** (s1701-1-al1), 507 bp.
TTAACTCCCAACCCCTGTGACATACCTTAATGTTGCCTCGGCGGATCAGCCCGCGCCCCGTAAAACGGGACGGCCC
GCCAGAGGACCCAAACTCTAATGTTTCTTATTGTAACTTCTGAGTAAAACAAACAAATAAATCAAAACTTTCAACAA
CGGATCTCTTGGTTCTGGCATCGATGAAGAACGCAGCAAAATGCGATAAGTAATGTGAATTGCAGAATTCAGTGA
ATCATCGAATCTTTGAACGCACATTGCGCCCGCTGGTATTCCGGCGGGCATGCCTGTTCGAGCGTCATTTCAACCCT
CAAGCCCCCGGGTTTGGTGTTGGGGATCGGCTCTGCCTTCTGGCGGTGCCGCCCCCGAAATACATTGGCGGTCTC
GCTGCAGCCTCCATTGCGTAGTAGCTAACACCTCGCAACTGGAACGCGGCGCGGCCATGCCGTAAAACCCCAACTT
CTGAATGTTGACCTCGGATCAGGTAGGAATACCCGCTGAACTTAAGCATAT

Fusarium tricinctum, MT446111.1, 534 bp., Percent identity 100%
TTTCCGAGTTAACTCCCAACCCCTGTGACATACCTTAATGTTGCCTCGGCGGATCAGCCCGCGCCCCGTAAAACGG
GACGGCCCGCCAGAGGACCCAAACTCTAATGTTTCTTATTGTAACTTCTGAGTAAAACAAACAAATAAATCAAAAC
TTTCAACAACGGATCTCTTGGTTCTGGCATCGATGAAGAACGCAGCAAAATGCGATAAGTAATGTGAATTGCAGA
ATTCAGTGAATCATCGAATCTTTGAACGCACATTGCGCCCGCTGGTATTCCGGCGGGCATGCCTGTTCGAGCGTCA
TTTCAACCCTCAAGCCCCCGGGTTTGGTGTTGGGGATCGGCTCTGCCTTCTGGCGGTGCCGCCCCCGAAATACATT
GGCGGTCTCGCTGCAGCCTCCATTGCGTAGTAGCTAACACCTCGCAACTGGAACGCGGCGCGGCCATGCCGTAAA

ACCCCAACTTCTGAATGTTGACCTCGGATCAGGTAGGAATACCCGCTGAACTTAAGCATATCAATAAGCCGGAGG
AAAAA

14. ***Trichoderma* sp., MZ427928** (s1702-3-al8), 585 bp.
TCATTACCGAGTTTACAACTCCCAAACCCAATGTGAACGTTACCAAACTGTTGCCTCGGCGGGATCTCTGCCCCGG
GTGCGTCGCAGCCCCGGACCAAGGCGCCCGCCGGAGGACCAACCAAAACTCTTTTTGTATACCCCCTCGCGGGTTT
TTTATAATCTGAGCCTTCTCGGCGCCTCTCGTAGGCGTTTCGAAAATGAATCAAAACTTTCAACAACGGATCTCTTG
GTTCTGGCATCGATGAAGAACGCAGCGAAATGCGATAAGTAATGTGAATTGCAGAATTCAGTGAATCATCGAATC
TTTGAACGCACATTGCGCCCGCCAGTATTCTGGCGGGCATGCCTGTCCGAGCGTCATTTCAACCCTCGAACCCCTCC
GGGGGGTCGGCGTTGGGGATCGGCCCTGCCTTGCCGGTGGACGTCTGCGAGATACAGTGGCGGTCTCGCCGCAG
CCTCTCCTGCGCAGTAGTTTGCACACTCGCATCGGGAGCGCGGCGCGTCCACAGCCGTTAAACACCCAACTTCAGA
AATGTTGACCTCGGATCAGGTAGGAATACCCGCTGAACTTAACCATATCAATAA

Trichoderma harzianum, MT422092.1, 621bp., Percent identity 98.97%
TTCCCCGTAGGTGAACCTGCGGAGGGATCATTACCGAGTTTACAACTCCCAAACCCAATGTGAACGTTACCAAACT
GTTGCCTCGGCGGGATCTCTGCCCCGGGTGCGTCGCAGCCCCGGACCAAGGCGCCCGCCGGAGGACCAACCAAA
ACTCTTTTTGTATACCCCCTCGCGGGTTTTTTATAATCTGAGCCTTCTCGGCGCCTCTCGTAGGCGTTTCGAAAATGA
ATCAAAACTTTCAACAACGGATCTCTTGGTTCTGGCATCGATGAAGAACGCAGCGAAATGCGATAAGTAATGTGA
ATTGCAGAATTCAGTGAATCATCGAATCTTTGAACGCACATTGCGCCCGCCAGTATTCTGGCGGGCATGCCTGTCC
GAGCGTCATTTCAACCCTCGAACCCCTCCGGGGGGTCGGCGTTGGGGATCGGCCCTGCCTTGGCGGTGGCCGTCT
CCGAAATACAGTGGCGGTCTCGCCGCAGCCTCTCCTGCGCAGTAGTTTGCACACTCGCATCGGGAGCGCGGCGCG
TCCACAGCCGTTAAACACCCAACTTCTGAAATGTTGACCTCGGATCAGGTAGGAATACCCGCTGAACTTAAGCATA
TCAATAAGCGGAGGAA
